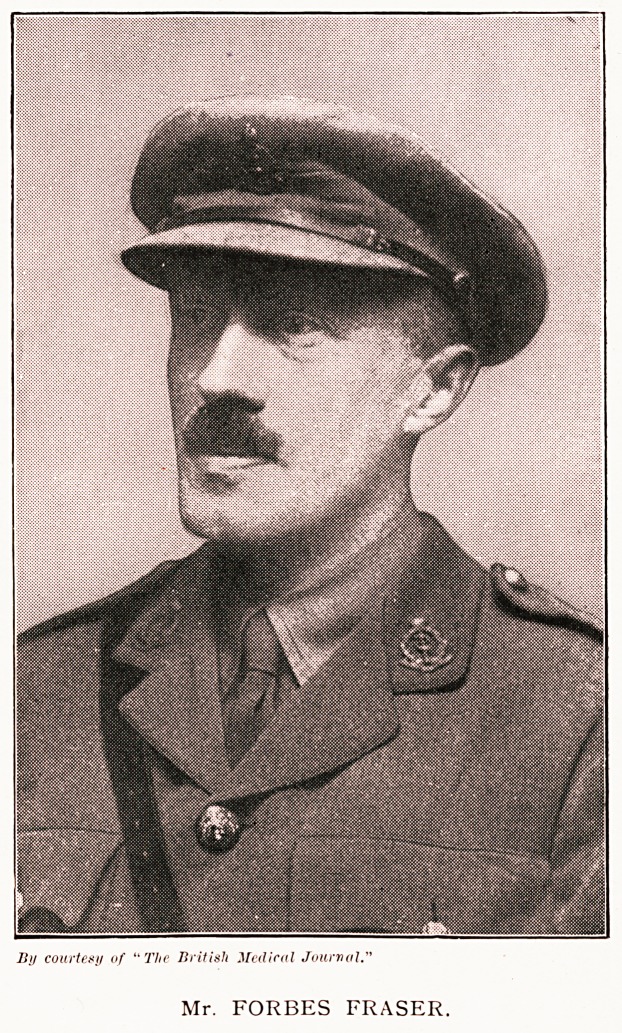# Forbes Fraser

**Published:** 1924-07

**Authors:** 


*By courtesy of "The British Medical Journal."*

Mr. FORBES FRASER.

### FORBES FRASER, C.B.E., F.R.C.S.

The death of Forbes Fraser on May 28th, at the early age of 53, was a sad loss to the profession and particularly to his colleagues in the West of England. His long illness was caused by a finger-prick whilst operating, and was borne with a fortitude which occasioned no surprise to those who knew him well.

Fraser was born in 1871, the younger son of Mr. Henry Fraser, of Arbroath. At the age of 16 he passed the London matriculation and gained an entrance scholarship at St. Bartholomew's Hospital. He won many other prizes and scholarships as a medical student, including the gold medal and an exhibition for Physiology at the intermediate London M.B. examination. He obtained the conjoint diplomas in 1894 and the F.R.C.S. Eng. in 1896. After qualifying he became Sir Henry Butlin's house surgeon at Barts. Then for three years he practised at Tarporley in Cheshire before he finally settled (in 1900) at Bath as the partner of Mr. Pagan Lowe. In 1903 he was appointed Assistant Surgeon at the Royal United Hospital, Bath, and in 1909 full Surgeon. At the time of his death he was Senior Surgeon at the Royal United. He was also Consulting Surgeon to the Ministry of Pensions Hospital, Bath, and to the hospitals at Chippenham, Frome, Malmesbury and Shepton Mallet.

During the war Fraser joined the Duchess of Westminster's Hospital at Le Touquet as surgeon, where he was a general favourite and won universal recognition for his surgical talents. In 1917 he left the Duchess of Westminster's Hospital and became a Captain in the R.A.M.C. He was selected to take up special work at No. 10 Casualty Clearing Station in the Second Army in the Ypres Salient. The results of this work were published in *The British Journal of Surgery* under the title "Primary and Delayed Primary Suture of Gunshot Wounds." During 1917 he was kept busy in the deplorable Passchendaele offensive. In 1918 he was chiefly engaged in the various C.C.S.'s of the Fourth Army on the Somme. He was an untiring worker and stood the strain of battle-surgery with marvellous endurance. His radient smile and cheery voice acted as a tonic on all who worked beside him.

Towards the end of 1918 he was appointed Consulting Surgeon to the Second Army, and after the Armistice he advanced with this force into Germany, where its title was changed to the Army of the Rhine, and he remained in Cologne as its consulting surgeon. He retired from the army with the rank of Colonel A.M.S. and the C.B.E. (military).

On his return to civil practice in Bath he found new work awaiting him, into which he threw himself with all his energy. He realised the need of the community for extended hospital accommodation and facilities, and set out to plan a scheme which should link up well-equipped cottage hospitals and hospitals in smaller towns with the Royal United as a consultative centre. Fraser's persuasive manner and tactful forbearance disarmed opposition and overcame indifference, so that he finally won the enthusiastic support of the profession and the public for his far-seeing schemes. It was a great satisfaction to him even in his last illness that the Duke of Connaught should have come to Bath on May 16th last to declare open the Royal United Private Hospital and Orthopædic Hospital at Combe Park, Bath. Mainly owing to Fraser the site of the Bath War Hospital was acquired by the Board of Management of the Royal United with the ultimate intention of transferring the whole hospital there.

Forbes Fraser took an active part in every side of professional life. In 1912–13 he was Chairman of the Bath Division of the Bath and Bristol Branch of the B.M.A., and in April last had been elected President-elect for the annual meeting of the British Medical Association in 1925 at Bath.

He had been President of the Bath Clinical Society, which he had helped to found. He was an active member and founder of various surgical clubs, among them the Country Surgeons' Club and the Bath and Bristol Surgical Club. He was a member of the Committee of the Bristol Medico-Chirurgical Society, where his communications and contributions to discussion were highly appreciated. The Society had hoped that he would have occupied the presidential chair in the near future.

It is difficult to express adequately the affection and admiration that Forbes Fraser inspired. As one of his colleagues wrote in the *British Medical Journal,* "Gifted with good physique and a brilliant intellect, he carried out whatever he set himself to do with indomitable will and unsparing energy, and when he decided at the end of the war to confine his work to consulting and operative surgery, he threw himself into his task with whole-hearted devotion and unbounded enthusiasm." His colleague at Le Touquet, Dr. W. P. S. Branson, summed him up when he wrote, "He was a distinguished man by the evidence of his material achievements, but what really stamped him as being beyond the common run of men was the blending in him of so many graces of spirit."

One is reminded of words written by the poet Thomas Gray: "Some spirit, something of a genius (more than common), is required to teach a man how to employ himself."

Forbes Fraser was twice married. By his first marriage he leaves two sons and three daughters. By the second marriage there is one daughter. His second wife survives him, and to her and all his family we extend our deep sympathy.

## Figures and Tables

**Figure f1:**